# Distinguishing grade I meningioma from higher grade meningiomas without biopsy

**DOI:** 10.18632/oncotarget.5376

**Published:** 2015-10-09

**Authors:** John Varlotto, John Flickinger, Martin T. Pavelic, Charles S. Specht, Jonas M. Sheehan, Dana T. Timek, Michael J. Glantz, Steven Sogge, Christopher Dimaio, Richard Moser, Shakeeb Yunus, Thomas J. Fitzgerald, Urvashi Upadhyay, Paul Rava, Matthew Tangel, Aaron Yao, Sangam Kanekar

**Affiliations:** ^1^ University of Massachusetts Medical Center, Department of Radiation Oncology, Worcester, MA, USA; ^2^ University of Pittsburgh Medical Center, Department of Radiation Oncology, Pittsburgh, PA, USA; ^3^ Columbia University Medical Center, Department of Anesthesia, New York, NY, USA; ^4^ Penn State Hershey Medical Center, Department of Pathology, Hershey, PA, USA; ^5^ Penn State Hershey Medical Center, Department of Neurosurgery, Hershey, PA, USA; ^6^ Penn State Hershey Neuroscience Institute, Hershey, PA, USA; ^7^ Penn State Hershey Medical Center, Department of Radiology, Hershey, PA, USA; ^8^ Penn State Hershey Medical Center, Department of Neurology, Hershey, PA, USA; ^9^ University of Massachusetts Medical Center, Division of Neurosurgery, Worcester, MA, USA; ^10^ University of Massachusetts Medical Center, Department of Medical Oncology, Worcester, MA, USA; ^11^ Penn State College of Medicine, Hershey, PA, USA; ^12^ Department of Healthcare Policy and Research, Virginia Commonwealth University, Richmond, VA, USA

**Keywords:** meningioma, MRI, cerebrovascular accident, tumor vascularity

## Abstract

**Background:**

Many meningiomas are identified by imaging and followed, with an assumption that they are WHO Grade I tumors. The purpose of our investigation is to find clinical or imaging predictors of WHO Grade II/III tumors to distinguish them from Grade I meningiomas.

**Methods:**

Patients with a pathologic diagnosis of meningioma from 2002–2009 were included if they had pre-operative MRI studies and pathology for review. A Neuro-Pathologist reviewed and classified all tumors by WHO 2007. All Brain MRI imaging was reviewed by a Neuro-radiologist. Pathology and Radiology reviews were blinded from each other and clinical course. Recursive partitioning was used to create predictive models for identifying meningioma grades.

**Results:**

Factors significantly correlating with a diagnosis of WHO Grade II-III tumors in univariate analysis: prior CVA (*p* = 0.005), CABG (*p* = 0.010), paresis (*p* = 0.008), vascularity index = 4/4: (*p* = 0.009), convexity vs other (*p* = 0.014), metabolic syndrome (*p* = 0.025), non-skull base (*p* = 0.041) and non-postmenopausal female (*p* = 0.045). Recursive partitioning analysis identified four categories: 1. prior CVA, 2. vascular index (vi) = 4 (no CVA), 3. premenopausal or male, vi < 4, no CVA. 4. Postmenopausal, vi < 4, no CVA with corresponding rates of 73, 54, 35 and 10% of being Grade II-III meningiomas.

**Conclusions:**

Meningioma patients with prior CVA and those grade 4/4 vascularity are the most likely to have WHO Grade II-III tumors while post-menopausal women without these features are the most likely to have Grade I meningiomas. Further study of the associations of clinical and imaging factors with grade and clinical behavior are needed to better predict behavior of these tumors without biopsy.

## INTRODUCTION

Meningiomas are the most common non-glial brain tumors, representing 15–25% of all primary brain tumors [1]. Historically, 90–95% of meningiomas were classified as benign lesions, while the other 5–10% of tumors were classified as the more aggressive sub-types, atypical (Grade II) and anaplastic (Grade III) [2]. The WHO first classified meningioma in 1993. Using that criteria, even completed resected benign lesions had relatively high recurrence rates, approaching 20% at ten years [3]. Pathologists have subsequently tried to re-classify lesions in order to better predict biologic behavior. In 2000, the WHO grading system was updated using more objective criteria and was determined to be more reproducible [4]. Grade II and III lesions were determined by number of mitosis per ten high-powered fields (≥4), high-risk sub-type (clear cell, choroid, papillary, and rhabdoid), and high-risk histologic features (three of the following five: small cell change, increased cellularity, prominent nucleoli, sheet-like growth or necrosis). In 2007, the WHO grading schema was modified with the major difference being that brain invasion could be used to classify otherwise grade I meningiomas as grade II. [5] One recent study evaluated all three pathologic classification systems in 196 specimens and demonstrated that the percentage of Grade II/III tumors increased with each consecutive pathologic staging system from 19.9% to 26.5% and to 31.1% respectively [6].

Many meningiomas are located at the skull base or other high-risk areas that do not facilitate easy surgical access for resection or even biopsy. Many other meningiomas occur in patients who are poor medical risk for resection or biopsy. Since many such meningioma are observed or irradiated without tissue diagnosis, we feel that a non-invasive approach to predict meningioma grade would be a highly beneficial management guide for these patients. The purpose of our study is to investigate whether MRI parameters, patient factors, co-morbidities, and/or medications could be used to predict lesion aggressiveness. All lesions were reviewed by a Neuroradiologist and a Neuropathologist in a blinded fashion from each other and from clinical outcomes. Lesions presenting prior to 2007 were re-classified according to the WHO 2007 grading schema.

## MATERIALS AND METHODS

After obtaining IRB approval (IRB#32319EP), we identified meningioma patients through our tumor registry and by specimen records in the Pathology Department at Penn State Hershey Medical Center from 2002–2009. We originally identified 100 cases, but only 85 patients had pathologic specimens and MRI imaging available for review. A board-certified Neuropathologist and a Pathology resident (CS, DT) reviewed all pathology specimens using WHO 2007 criteria completely without knowledge of clinical outcome or radiological imaging characteristics. Specimens were classified by grade, sub-type, brain invasion, bone invasion, cellularity, nuclear/cytoplasmic ratio, prominent nucleoli, sheet-like growth pattern, and foci of necrosis.

We reviewed medical charts to record patient factors including medications, symptoms co-morbidities race, age, body mass index, pregnancy, smoking/alcohol history, and sex into a de-identified database. Symptoms analyzed included seizures, memory loss, visual loss/disturbance, headaches, nausea, vomiting, cranial neuropathy, hearing loss, ataxia, and paresthesias. Medical conditions analyzed included past therapeutic radiation exposure (external beam/srs to tumor or delivery to areas outside the CNS), osteoporosis, diabetes, hypertension, past myocardial infarction, cardiac arrhythmia, renal failure, thromboembolic disease, CVA, and Charlson co-morbidity index [7]. No patients in our series had a history of NF-1 or NF-2. Medications analyzed included aspirin, statins, NSAIDS, and hormonal therapy (estrogen replacement). No patients were noted to be taking 5-alpha reductase inhibitors, tamoxifen or oral contraceptive pills at the time of diagnosis.

**Figure 1 a-d:**
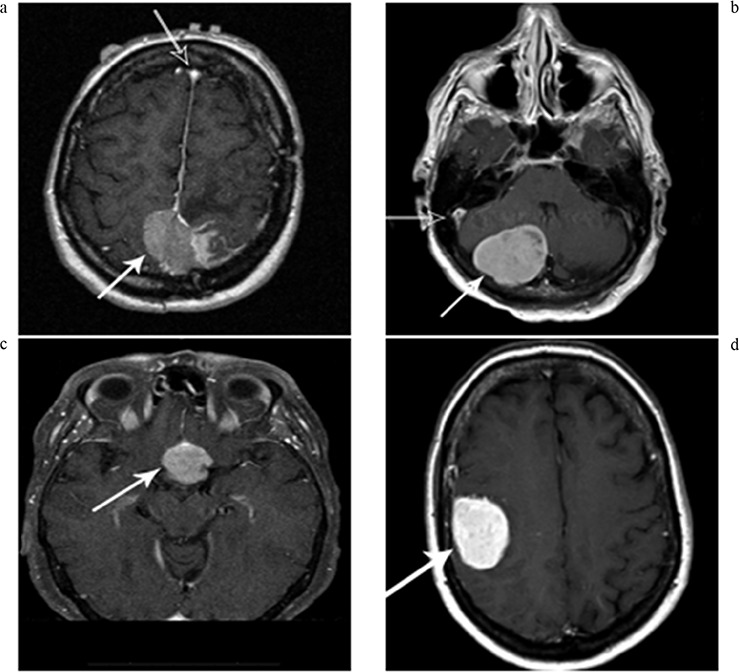
Tumor vascularity as noted by the degree of tumor enhancement A = Vascularity 1- Baseline vascularity; defined by markedly low signal (white arrow) compared to the dural venous sinuses (black arrow), typically the superior sagittal sinus, B = Vascularity 2–Mild enhancement, but low signal compared to the superior sagittal sinus, C = Vascularity 3–moderate enhancement with signal equal to the superior sagittal sinus and pituitary gland, D = Vascularity 4–Avidly enhancing tumor with high signal compared to the superior sagittal sinus.

85 of the 92 patients with available pathology had MRI Scans available for re-evaluation. MRI scans were analyzed for location, brain invasion, bone invasion, brain herniation, edema, hyperostosis, necrosis, vessel invasion, hemorrhage, calcification, cystic change, vascularity index, tumor volume, brain herniation/midline shift, and number of tumors. The vascularity index used a four-point grading system that was devised by the Neuro-Radiologists at Penn State Hershey Medical Center as noted by: Vascularity 1- Baseline vascularity, defined by markedly low signal compared to the dural venous sinuses, typically the superior sagittal sinus; Vascularity 2–Mild enhancement, but low signal compared to the superior sagittal sinus; Vascularity 3–moderate enhancement with signal equal to the superior sagittal sinus and pituitary gland; and Vascularity 4–Avidly enhancing tumor with high signal compared to the superior sagittal sinus. The four different grades for the vascular index can be seen in Figure 1.

**Figure 2:**
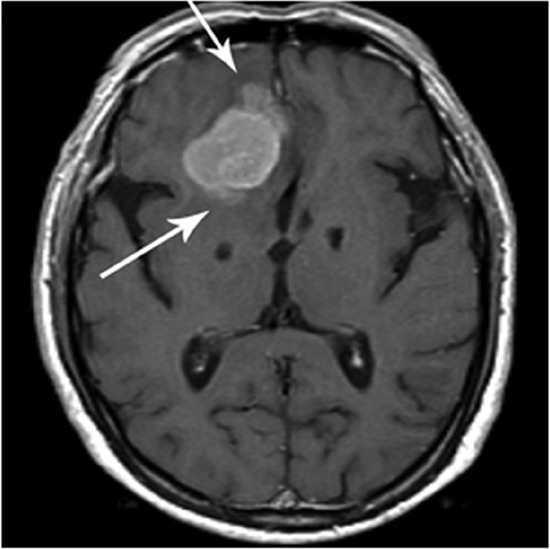
Brain invasion as noted by loss of fat plane (arrows) between tumor and normal brain

Radiologic brain invasion was noted by the loss of gray matter morphology and distortion as well as the loss of fat plane (arrows) between tumor and normal brain as noted in Figure 2.

Apparent diffusion coefficient was available in 44 of the 85 patients and classified as increased, decreased, mixed or same cellularity as normal brain.

Univariate analysis was performed of all patient characteristics, medications, symptoms, co-morbidities, and radiologic parameters to assess their association with grade I or grade II-III tumors. All factors in the univariate analysis with a *p*-value < 0.05 were analyzed by recursive partitioning analysis. Clusters of different genetic mutations have been identified among different locations and grades of meningiomas [8–10]. Because of this heterogeneity in meningiomas we felt that recursive partitioning analysis would be the best way to discern possible clusters of clinical and imaging characteristics to identify WHO Grade II-III meningiomas.

## RESULTS

### Pathology review

We identified 85 patients with both pathology and imaging available for review for this study. 54 (64%) were classified as WHO grade I while 31 (36%) were WHO Grade II-III; specifically with 29(34%) WHO-II and 2 (2%) WHO-III.

The four most common histological types were transitional (25%), meningothelial, (23%), atypical (22%), and fibrous (4%). Brain invasion was seen in 6%, and bone invasion in 6%, Increased cellularity was identified in 17%, high N/C ratio in 10%, prominent nucleoli in 27%, foci of necrosis in 28%, and uninterrupted pattern of sheet-like growth in 21% of specimens.

### Clinical characteristics

Most patients were female (68%) and white (81%). The median presenting age was 59.16 (range 2–86). The median BMI was 31.37 (obesity is BMI ≥30), with a range of 16.4–49. Only 3% of the patients were asymptomatic, and the most common presenting complaint was headaches that were reported in 35% of patients. Visual disturbances were reported in 25%, seizures in 23%, imbalance in 18%, memory loss in 17%, paresthesias in 16%, and cranial neuropathies in 15%. Prior radiation treatment to or near the head was reported in 5%. The most common co-morbidity was hypertension, seen in 56% of patients.

### Imaging review

Radiological/imaging review demonstrated that the most common location was the cerebral convexity, seen in 45% of patients. Other locations were: parasellar (6%), falx (6%), sphenoid ridge (3%), petroclinoid (3%), orbital/peri-orbital (2%), tentorial (2%), cerebellar pontine angle (2%), and other (16%). Median tumor volume was 10.13cc (median 5.48–12.56cc). Brain herniation/midline shift was seen in 45%. Vascularity of the tumors was graded with a vascularity index of 1, 2, 3, or 4 in 17%, 4%, 58% and 21% of the patients respectively.

We identified radiological brain invasion in 32%. 60% of tumors were associated with brain edema. Imaging evidence of necrosis was seen in 22%, blood vessel invasion in 10%, hyperostosis in 33%, bone invasion in 14%, hemorrhage in 4%, calcification in 24%, and cystic changes in 6%.

### Univariate analysis

Table 1 shows the clinical and imaging factors identified as significantly associated with Grade II/III by univariate analysis (*p* < 0.05) were: prior CVA73% WHO Grade II-III (8/11) vs 26% (18/68) without (*p* = 0.005), prior CABG surgery 100% WHO Grade II-III vs 29% without (*p* = 0.010), paresis 57% WHO II-III vs 23% without (*p* = 0.008), vascularity index = 4: 65% WHO Grade II-III vs 28% with vascularity index = 1–3 (*p* = 0.009), site: convexity 49% WHO Grade II-III vs 23% other (*p* = 0.014), metabolic syndrome 0/8 WHO Grade II-III vs 40% without (*p* = 0.025), site: skull base 13% WHO Grade II-III vs 42% other (*p* = 0.041) and postmenopausal female 23% yes vs 47% no (*p* = 0.045).

**Table 1:** Factors of univariate significance *p* < 0.05 (by chi-square or Fisher exact test) distinguishing WHO grade 1 versus WHO grade 2–3 meningiomas

Variables	% Gr 2–3 with variable Vs. without variable	*P*-value
Prior stroke	73% vs 26%	0.005
CABG	4/4 vs 29%	0.010
Paresis	57% vs 23%	0.008
Vascularity 4 (vs 1–3)	65% vs 28%	0.009
Site: Convexity	49% vs 23%	0.014
Metabolic Syndrome	0/8 vs 40%	0.025
Site: Skull Base	13% vs 42%	0.041
Post-menopausal Female	23% vs 47%	0.045

Borderline significance was seen for: multiple meningiomas (*p* = 0.060, 63% vs 32%), obesity with BMI >30 (*p* = 0.061, 22% vs 44%), and imaging defined brain invasion (*p* = 0.063, 50% vs 28%). Variables correlating with WHO Grade II-III at a level of 0.1 < *p* < 0.2 were: vessel invasion, coronary artery disease, NSAIDS, current oral contraceptive (OCP) use, hypertension, Vol > 17cc, and Pre-Menopausal Female. Variables with correlations to WHO Gr II-III at 0.2 < *p* < 0.5 were: symptomatic, Osteoporosis, presence of edema, current ocp use, statin use, cystic change, extracranial extension, memory loss, hyperostosis, site: tentorium, Smoking Never, past radiation exposure, paresthesia, cranial neuropathy, and current alcoholism. Variables with a correlation to WHO Grade II-III with *p* > 0.5 were: treated diabetes, aspirin use, history of alcoholism, nausea, hearing loss, diabetes known, gait ataxia, calcification, age > = 60, past-hormone replacement, brain herniation, and headaches.

### Recursive partitioning analysis

Table 2 lists the results of the recursive partitioning analysis which was performed using only the variables in Table 1.

**Table 2:** Recursive partitioning analysis (VI = vascularity index, Gr2–3 = WHO Grade 2–3)

Variables	% Gr 2–3 with variable Vs. without variable	P(Chi-square or Fisher)
Prior CVA: yes versus no	73% (8/11) vs 26% (18/68)	*p* = 0.005
A. Prior CVA	73% (8/11)	Subsets: 0.055 *< *p* <0.52
B. No prior CVA	26%(18/68)	
C. Vascularity = 4 vs 1–3	54% (7/13) vs 20% (11/54)	0.032
D. i. Vascularity 4	54% (7/13)	Subsets: 0.10 *< *p* <0.61
ii. Vascularity ≤3	20% (11/54)	
E. Post Menopausal:Y/N	10% (3/30) vs 35% (7/20)	*p* = 0.03
i. VI ≤3 Post Menopausal	10% (3/30)	
ii. VI ≤3 Other	35% (7/20)	
**Four Group Model**		
1. Prior CVA	73% (8/11)	
2. No CVA, VI = 4	54% (7/13)	
3. No CVA, VI ≤3, not PostMenopausal	35% (7/20)	
4. No CVA, VI ≤3, and PostMenopausal	10% (3/30)	

* Subset analysis: closest variable for CVA subset was Site: skull base 0/2 vs other 8/9 *p* = 0.055, closest subsets for no prior CVA and VI-4 were brain invasion by imaging and presence of other meningiomas, both *p* = 0.103.

The initial (most significant) partition identified the highest risk group as patients with a prior CVA where 73% (8/11) had WHO Grade II-III meningiomas. No significant further partitions were identified in that small highest-risk group. Among the meningioma patients with no prior CVA, the next (most significant) partition occurred with a vascular index (VI) of 4, where 54% (7/13) were WHO Grade II-III tumors and no significant further partitions were identified in the that group. Among the patients with no prior CVA and VI = 1–3, the next partition defined the group at lowest risk for WHO Grade II-III tumors as postmenopausal women (with no stroke and VI = 1–3) who had a 10% risk (3/30) of harboring WHO Grade II-III meningiomas compared to 35% (7/20) of meningioma patients with VI = 1–3 and no prior CVA who weren't postmenopausal females. The recursive partitioning analysis thus separated the patients into four risk groups with 73, 54, 35 and 10% risks of harboring WHO Gr II-III meningiomas. If the two highest risk groups are combined as shown in Table 2, then the three risk levels would be 63, 35 and 10%.

## DISCUSSION

To the best of our knowledge, our investigation is the first study to demonstrate that clinical factors (past history of a cerebrovascular accident and post-menopausal state) can be used in order to distinguish atypical/anaplastic meningiomas from the more common benign WHO Grade I meningiomas. A past report only assessed brain imaging characteristics and the correlation with higher grade tumors and demonstrated that intratumoral cystic change and extracranial tumor extension through the skull base foramina were more prevalent in atypical/malignant meningiomas [11]. However, unlike that report, our investigation had all pathologic specimens blindly reviewed by a Neuropathologist using the current 2007 WHO classification system. Additionally, we had almost twice as many higher-grade tumors which allowed us to perform a multivariate analysis.

Although our 36% rate of higher grade meningiomas may seem high, it should be noted that our percentage of higher grade tumors is similar to that of another recent series which noted that re-classification of meningiomas by the new WHO 2007 criteria was associated with a higher percentage of high grade tumors [6]. Additionally, we feel that the relatively high proportion of Grade II/III tumors may reflect a referral bias to our institution. Because past therapeutic radiation [12], BMI in females [13] and hormone replacement therapy [14] have been associated with the development of meningioma, we felt that assessing the association of these factors with the meningioma grade may be important. It should be noted that obesity is associated with higher adipose aromatase activity, estrogen, androgens, and insulin-like growth factor [15]. Despite the majority of our patients being female (68%) and obese (median BMI was 31.3), females were not more likely than males to be overweight (BMI >25, 78% vs 81%) or obese (BMI >30, 55% vs 50%). Somewhat surprising to us, we found a lower incidence of Grade II-III meningiomas in patients with metabolic syndrome (0/8 vs 40%, *p* = 0.024) with similar trends for just a diagnosis of obesity (23% vs 44%, *p* = 0.061). We were expecting to find higher rates of Grade II-III meningiomas in our premenopausal women than postmenopausal women (50% vs 26%), but were surprised to find the same 50% rate for the men in our series. If estrogen stimulates tumor growth, it is possible that it causes faster growing meningiomas to be diagnosed earlier and therefore before the onset of menopause. If the lower overall incidence of meningiomas in men than women included a greater decrease in the incidence of WHO Grade I than Grade II-III tumors, then the proportion of Grade II-III meningiomas would be higher in men than women. Because of the known carcinogenic properties of smoking, and the suspected anti-carcinogenic properties of statins [16] and cox-2 inhibitors [17], we decided to investigate these medications as well as hormonal therapy. For completion, we also investigated patient co-morbidities and presenting symptoms. We were surprised to find the highest rate of Grade II-III tumors in the small cohort of patients with prior strokes: 73% (8/11) Grade II-III vs 26% without (*p* = 0.005). We also found an association with CABG surgery (*p* = 0.003 on univariate analysis) and a trend for any coronary artery disease (*p* = 0.10). It is possible that factors that give rise to CVAs and coronary artery disease also lead to intratumoral hypoxia that in turn increases the chances of tumor mutation to higher grades. We hypothesize that hypoxia may act through the induction of hypoxia inducible factor-1 alpha thereby causing the up-regulation of pro-angiogenic genes [18]. Therefore, hypoxia in the surrounding brain tissue may provide a stimulating environment for higher grade characteristics to occur in meningioma. Of note, recently, high expression levels of VEGF were found in peri-necrotic tumors in Grade III meningiomas [19] and HIF-1 has been found in a greater percentage of higher grade meningiomas than Grade I tumors [20].

Our extensive analysis of imaging characteristics identified a significantly higher rates of Grade II-III meningiomas with a vascular index of 4/4 (54% vs 20%, *p* = 0.032). By univariate analysis, only tumor sites in the convexity (49% vs 23%) and non-skull base sites (42% vs 13%) were associated with higher grade tumors. Past studies have suggested that non-skull base locations [21, 22] were associated higher grade tumors. MRI is the mainstay of meningioma imaging because of its superior soft tissue resolution and multiplanar capabilities. To date, there has been no specific or reliable diagnostic feature with conventional or high-end MR imaging techniques for the differentiation of Grade II/III meningiomas from the more common low grade I tumors [23, 24]. Newer imaging techniques such as Diffusion-weighted (DWI), MR perfusion (MRP), Diffusion Tensor Imaging (DTI), and MR spectroscopy (MRS) have been investigated in smaller series with lesser success and conflicting results. [25–31]. Our study shows that the conventional MR imaging may be used to differentiate between benign and atypical/malignant meningiomas via grade 4/4 vascularity, but high grade vascularity is of lesser importance than previous history of CVA. As compared to the high end modalities like DTI, DWI, DWI and MRS, conventional structural imaging is routinely performed, less expensive, readily available, and easily applicable for evaluation of meningioma. Additionally, our technique for identifying Grade II-III meningiomas can allow centers to make decisions on patients without having to obtain expensive imaging studies that are unavailable from referring centers and possibly prevent biopsy in the growing proportion of elderly patients [32] who may undergo a brain MRI for other reasons.

The main weaknesses of this study are the difficulty of assessing so many clinical and imaging variables with a limited data set as well as the lack of genetic analysis of these tumors. Because of the large numbers of variables that were assessed in this limited database, this investigation was essentially an exploratory analysis that needs to be followed up via the use of a much larger database. Including data on genetic mutations in future studies would hopefully allow us to correlate specific mutations with these different clinical and imaging factors, but would even further compound the need for a larger database. Our hypothesis that different combinations of mutations would be associated with different combinations of clinical and imaging factors is why we chose recursive partitioning analysis as the next step to assess these variables after initial univariate analysis. The four risk categories we identified: 1. prior CVA, 2. vascular index(vi) = 4(no past CVA history), 3. premenopausal or male, vi < 4, no stroke, and 4. Postmenopausal, vi < 4, no past CVA history with 73, 54, 35 and 10% rates of harboring Grade II-III tumors seems a reasonable start towards characterizing these tumors based on clinical and imaging variables, but confirmation is needed in larger studies. Although long-term follow

## CONCLUSION

Meningioma patients with prior CVAs and with a grade 4/4 vascularity index are the most likely to have WHO Grade II-III tumors (73 & 54% respectively) while post-menopausal women without these features are the most likely to have WHO Grade I tumors (90%). Further study of the associations of clinical and imaging factors with grade and clinical behavior are needed to better predict behavior of these tumors without biopsy.
